# Supplementary figures and images for: Non-human peptides revealed in blood reflect the composition of intestinal microbiota
Source: BMC Biol. 2024 Aug 26;22:178. doi: 10.1186/s12915-024-01975-1 (PMC11346180; doi:10.1186/s12915-024-01975-1)

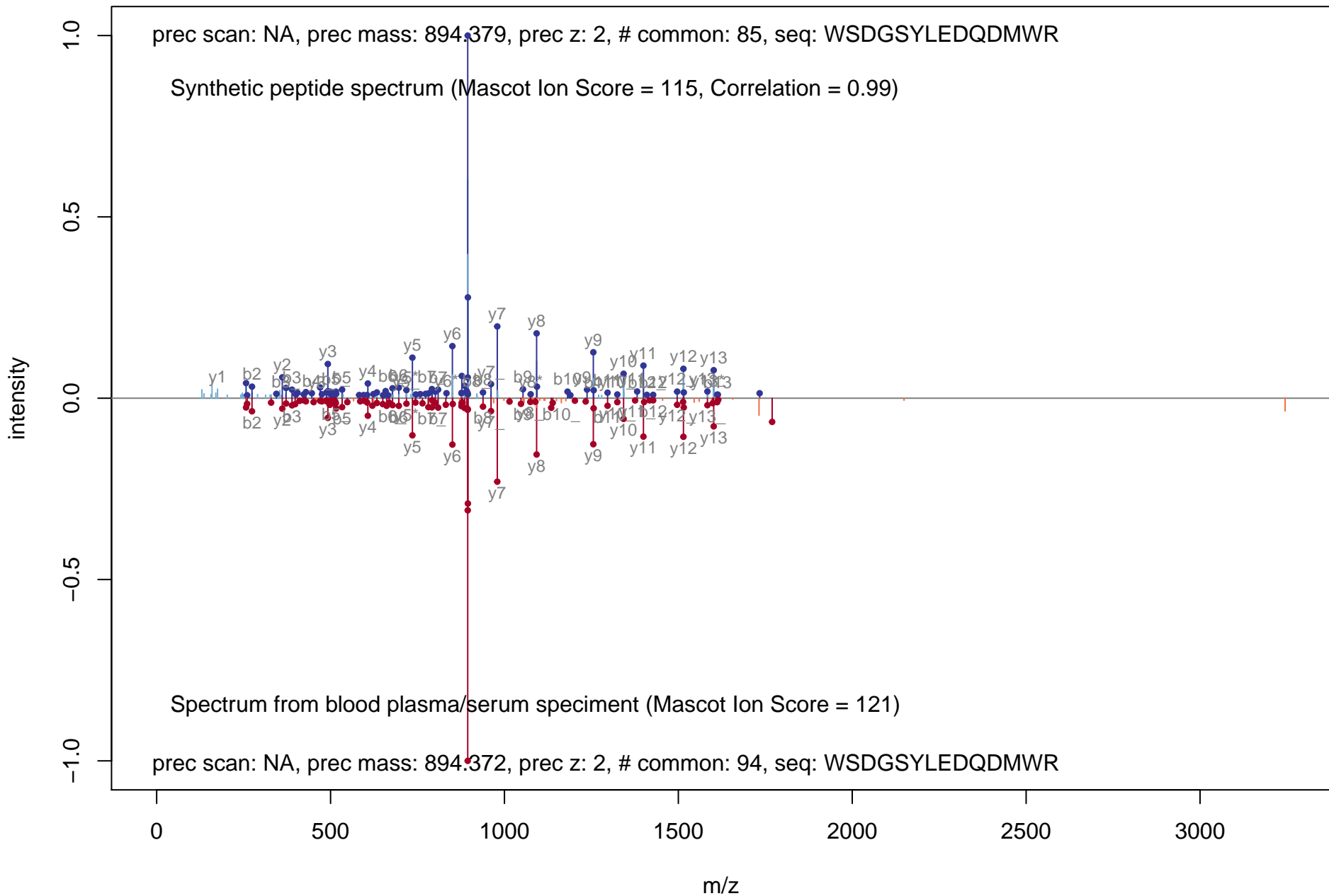

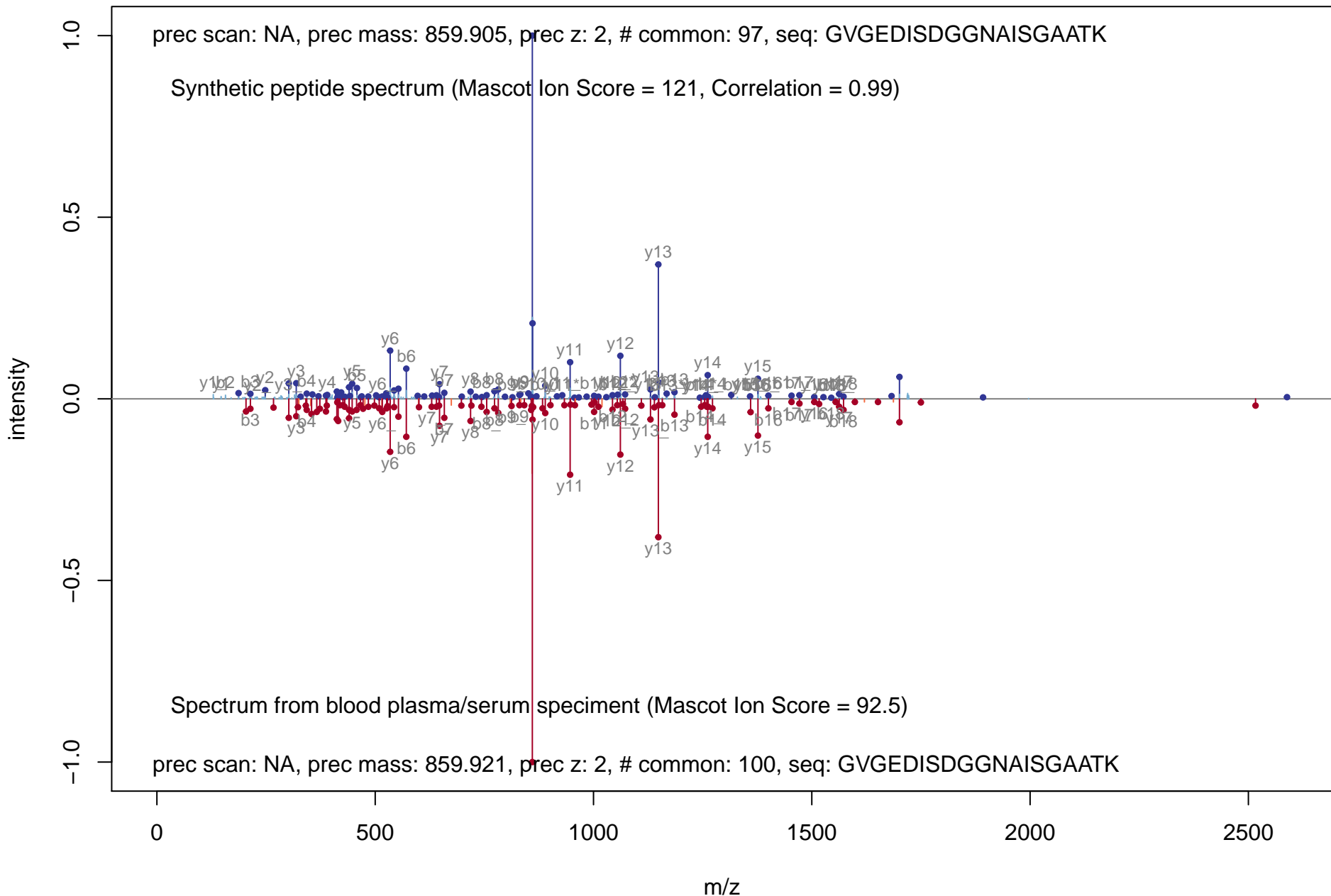

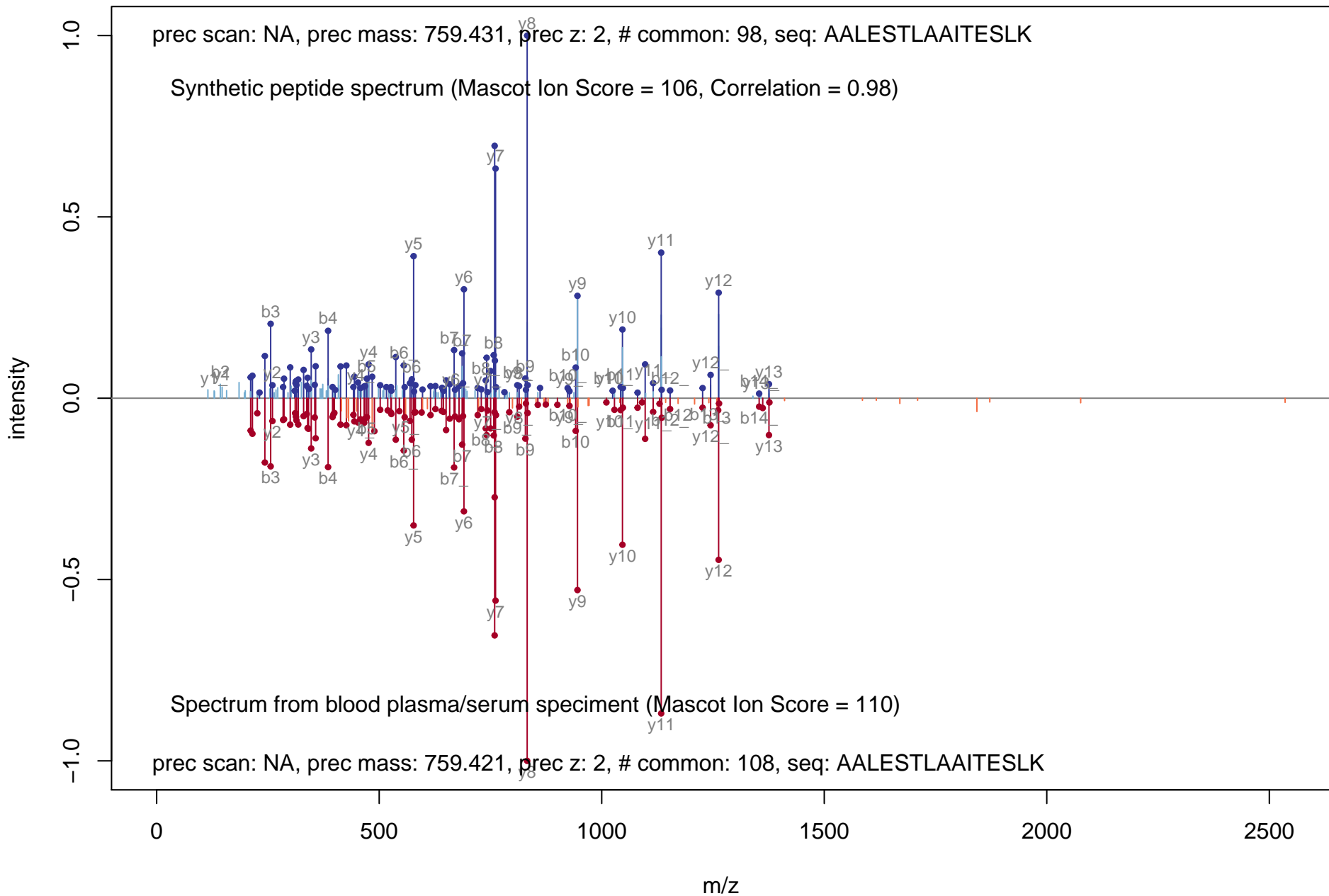

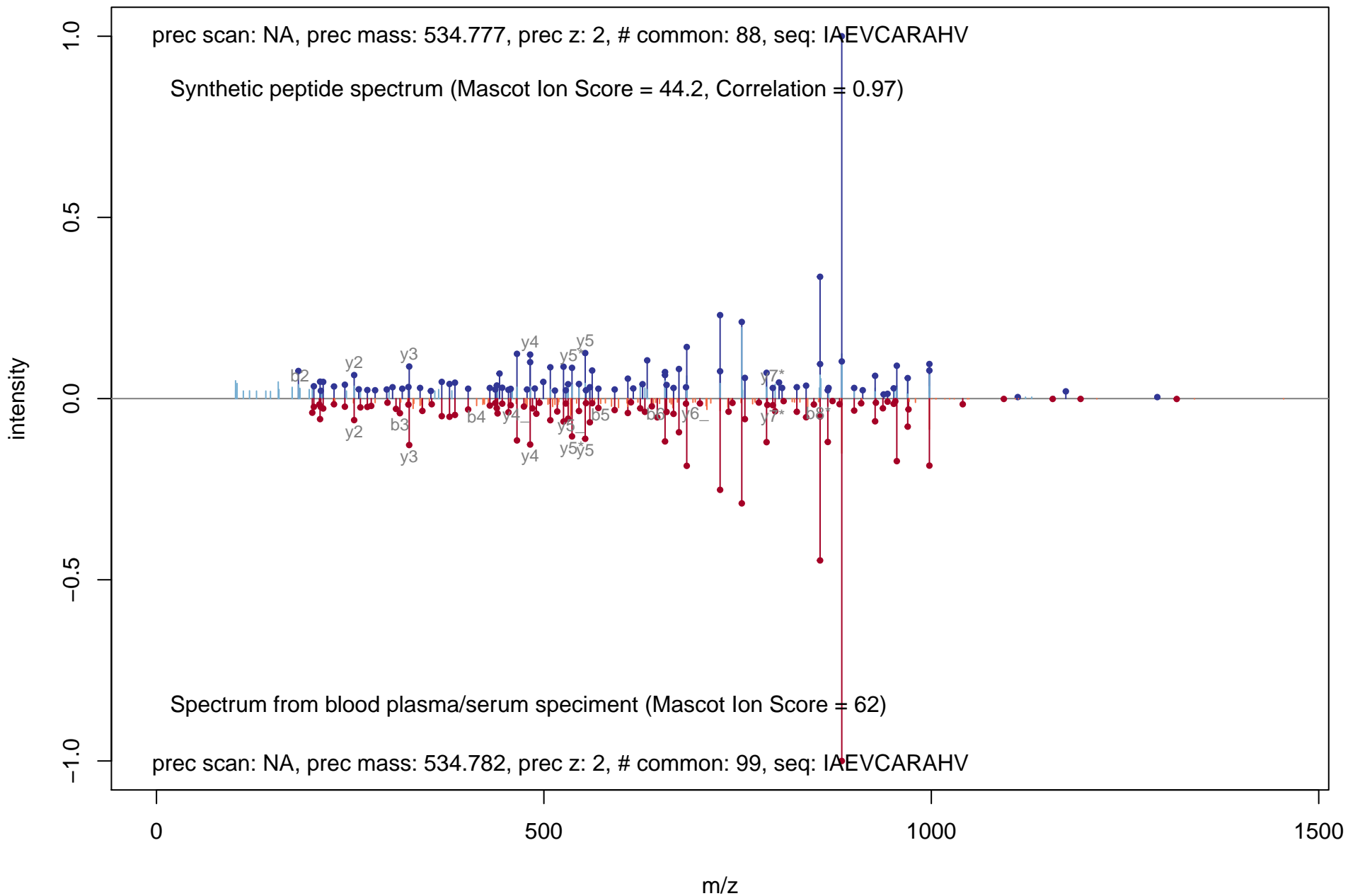

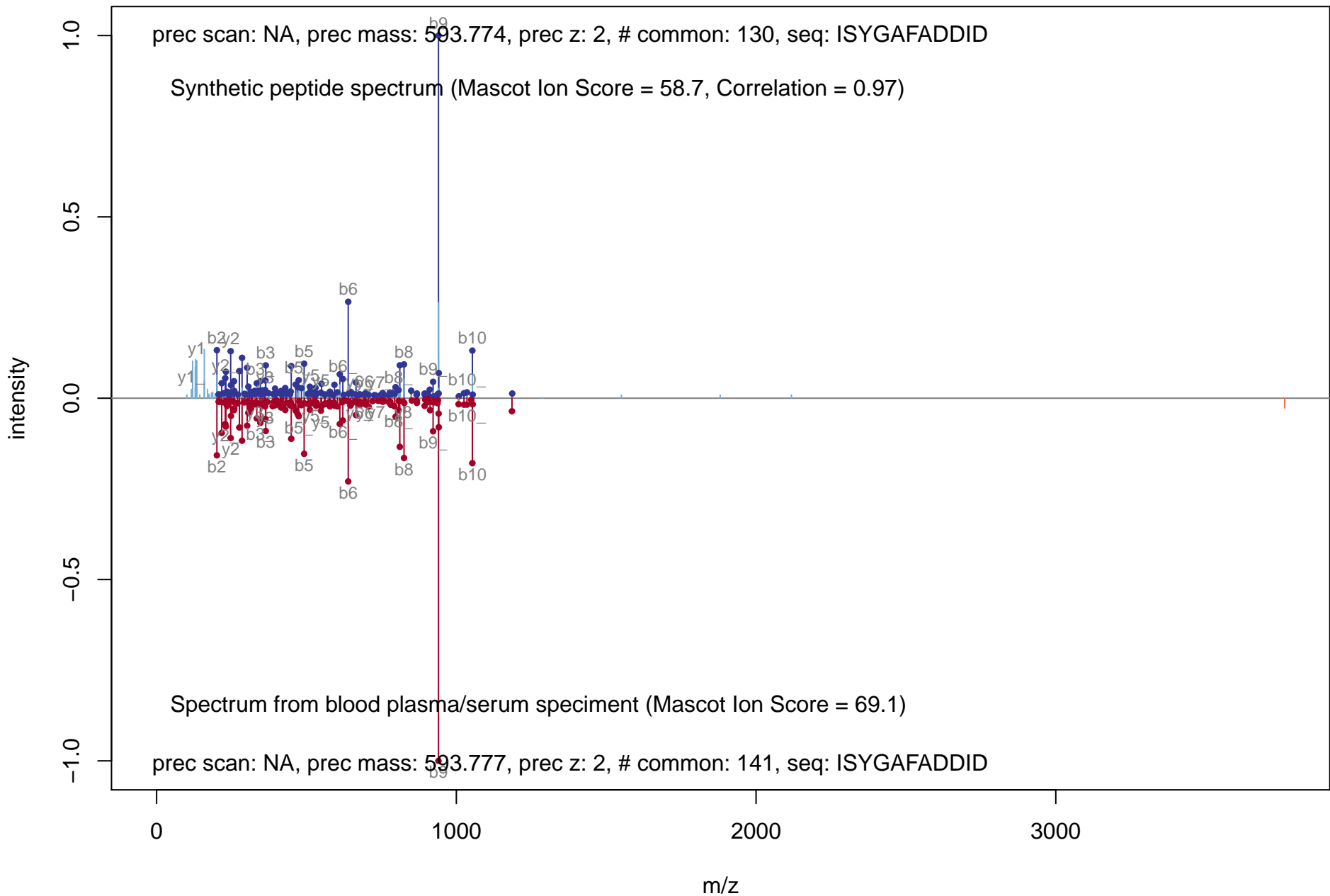

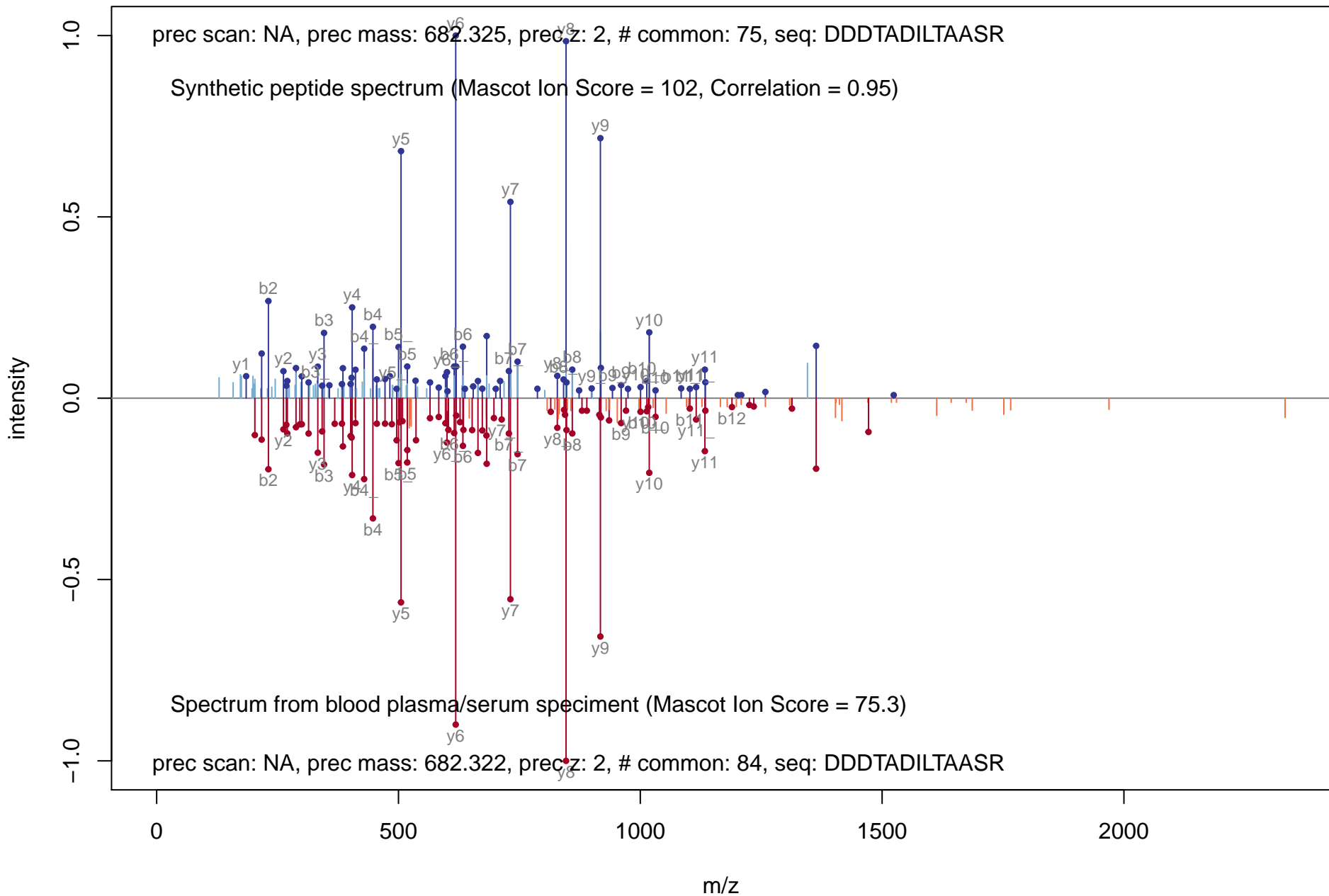

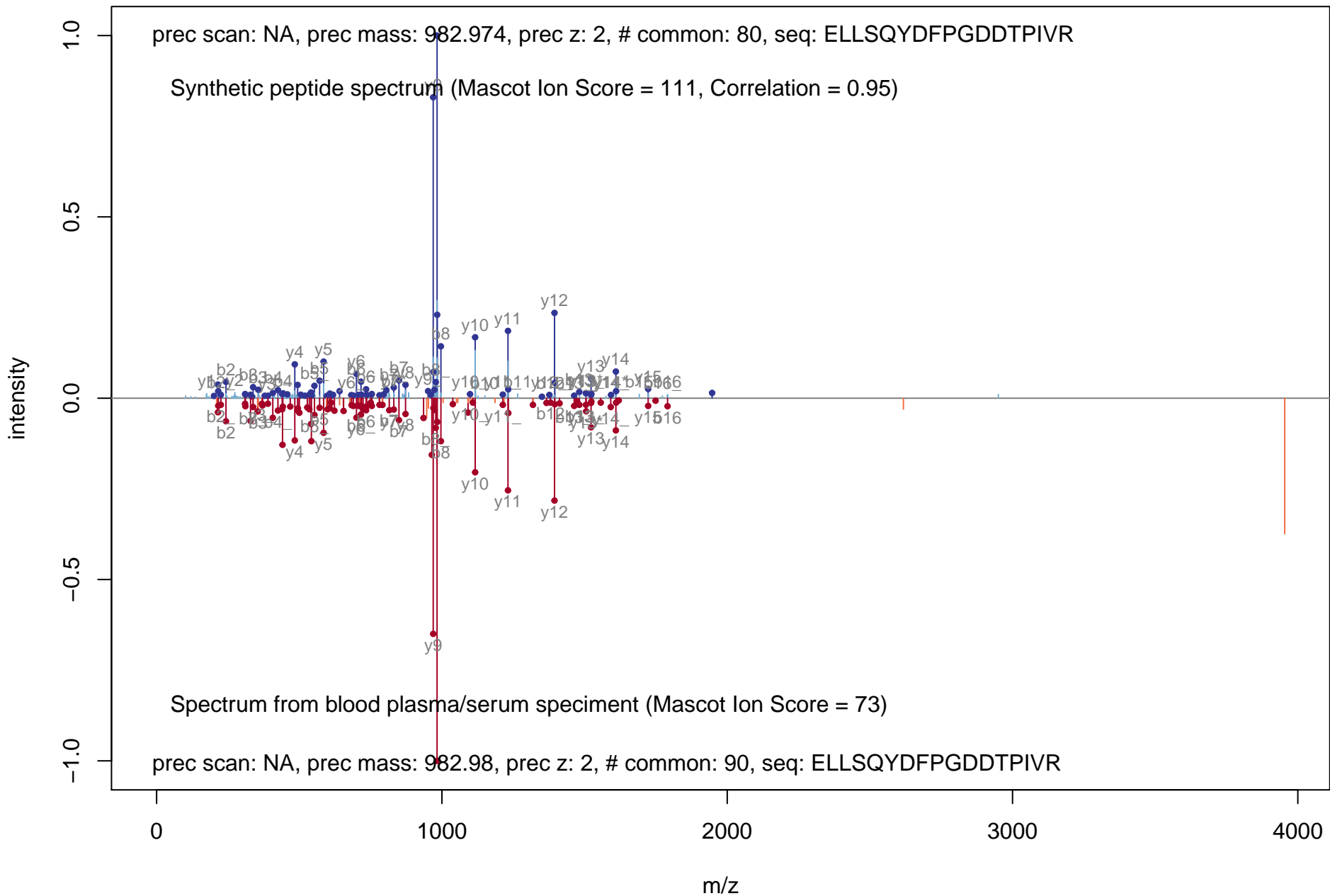

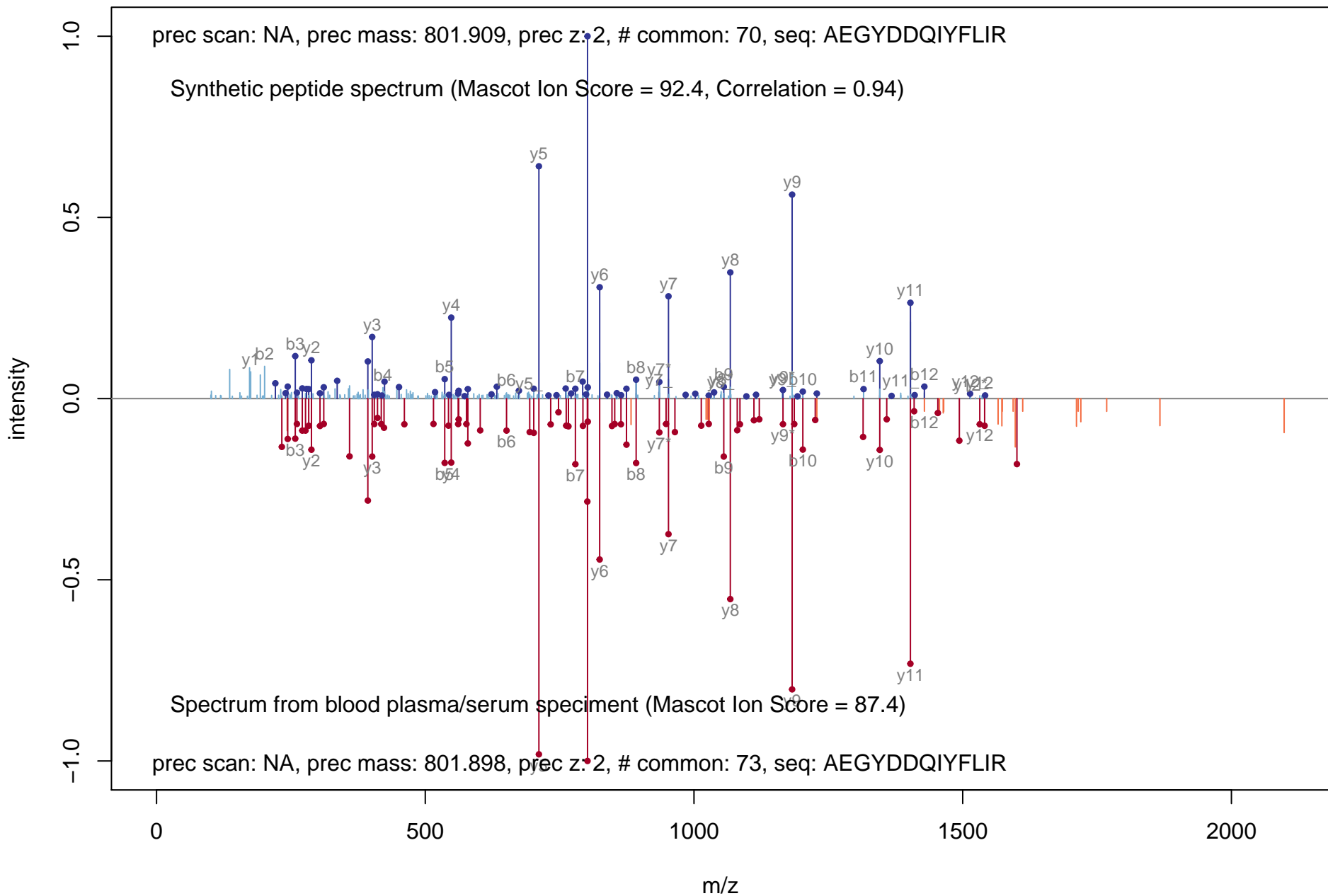

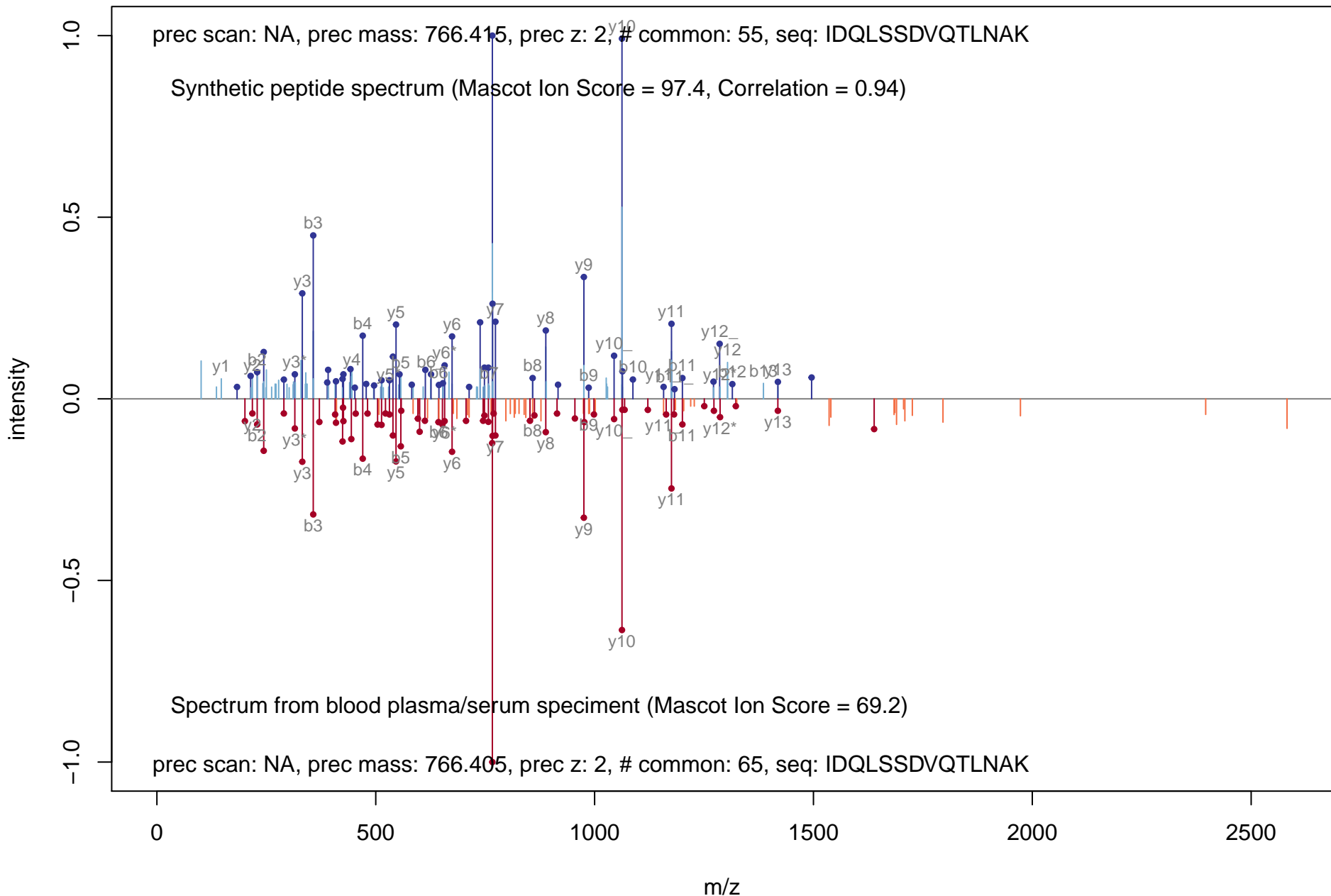

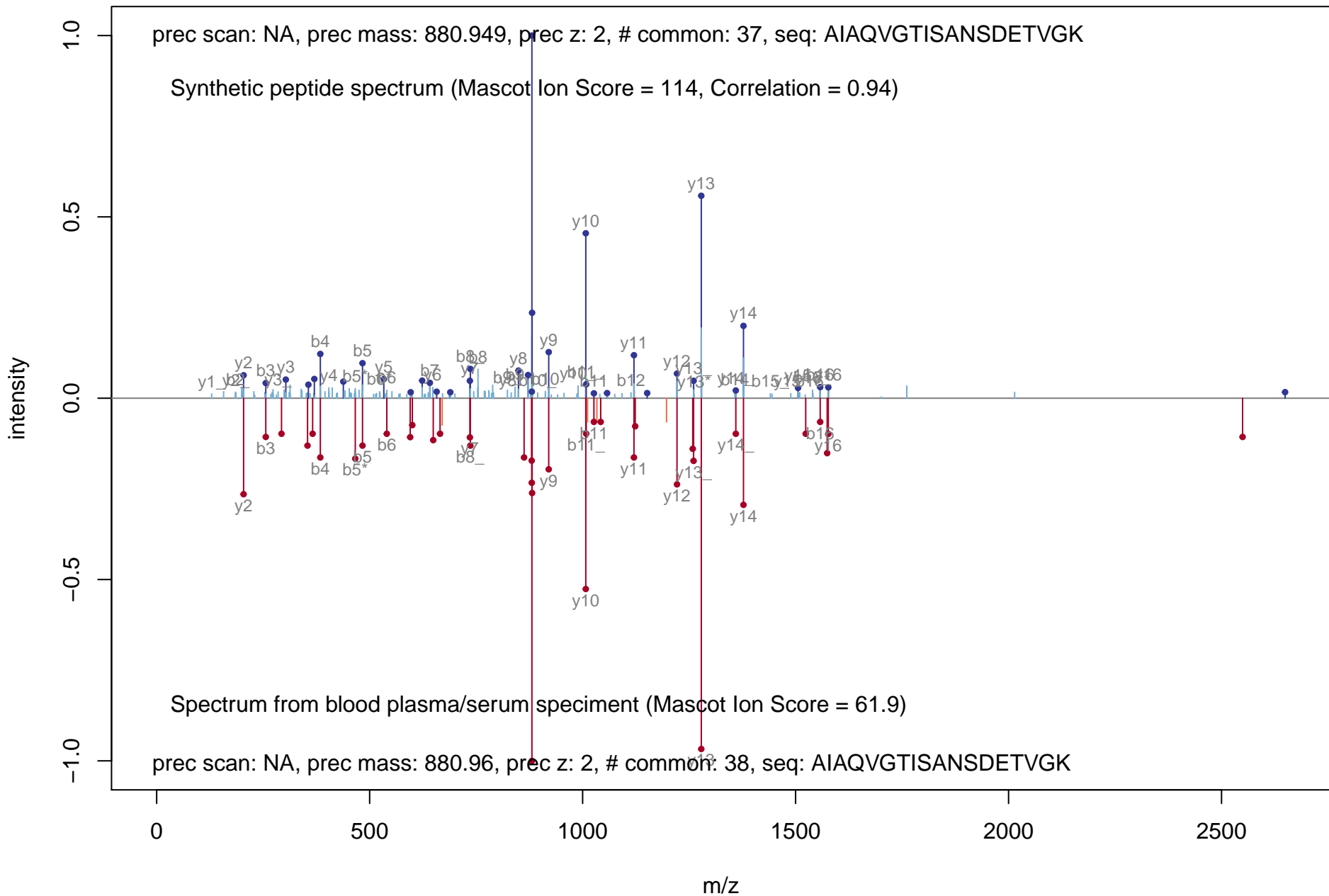

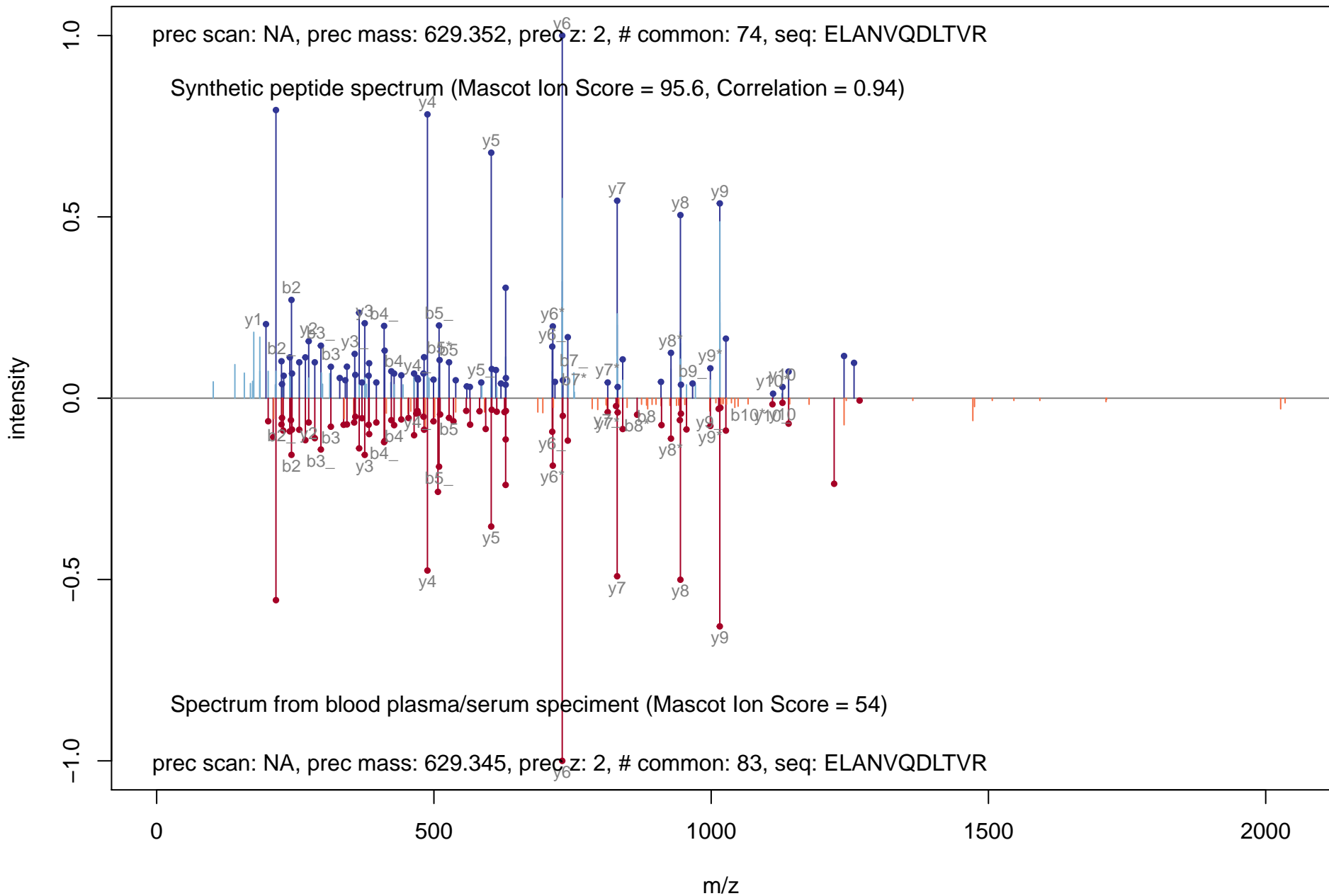

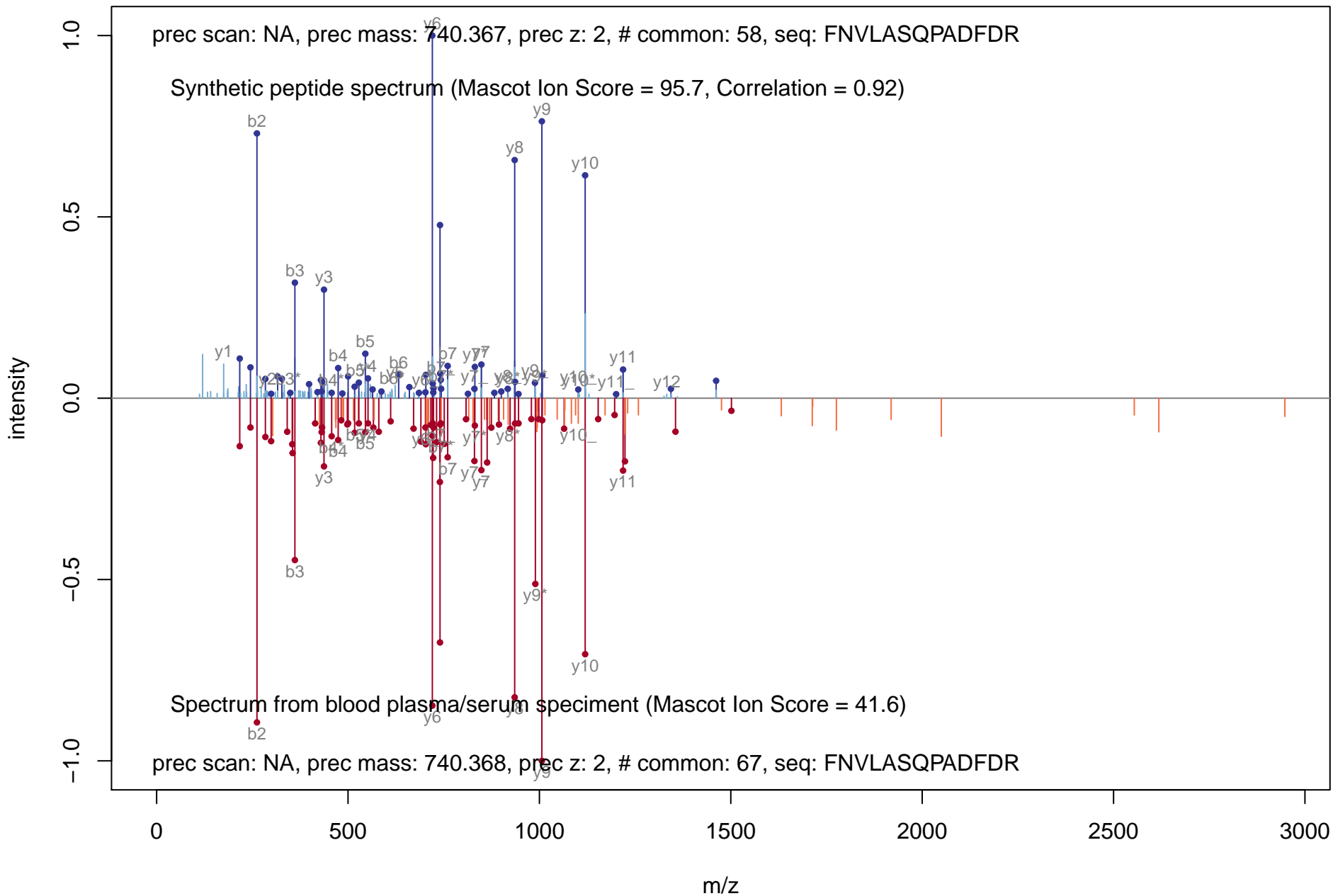

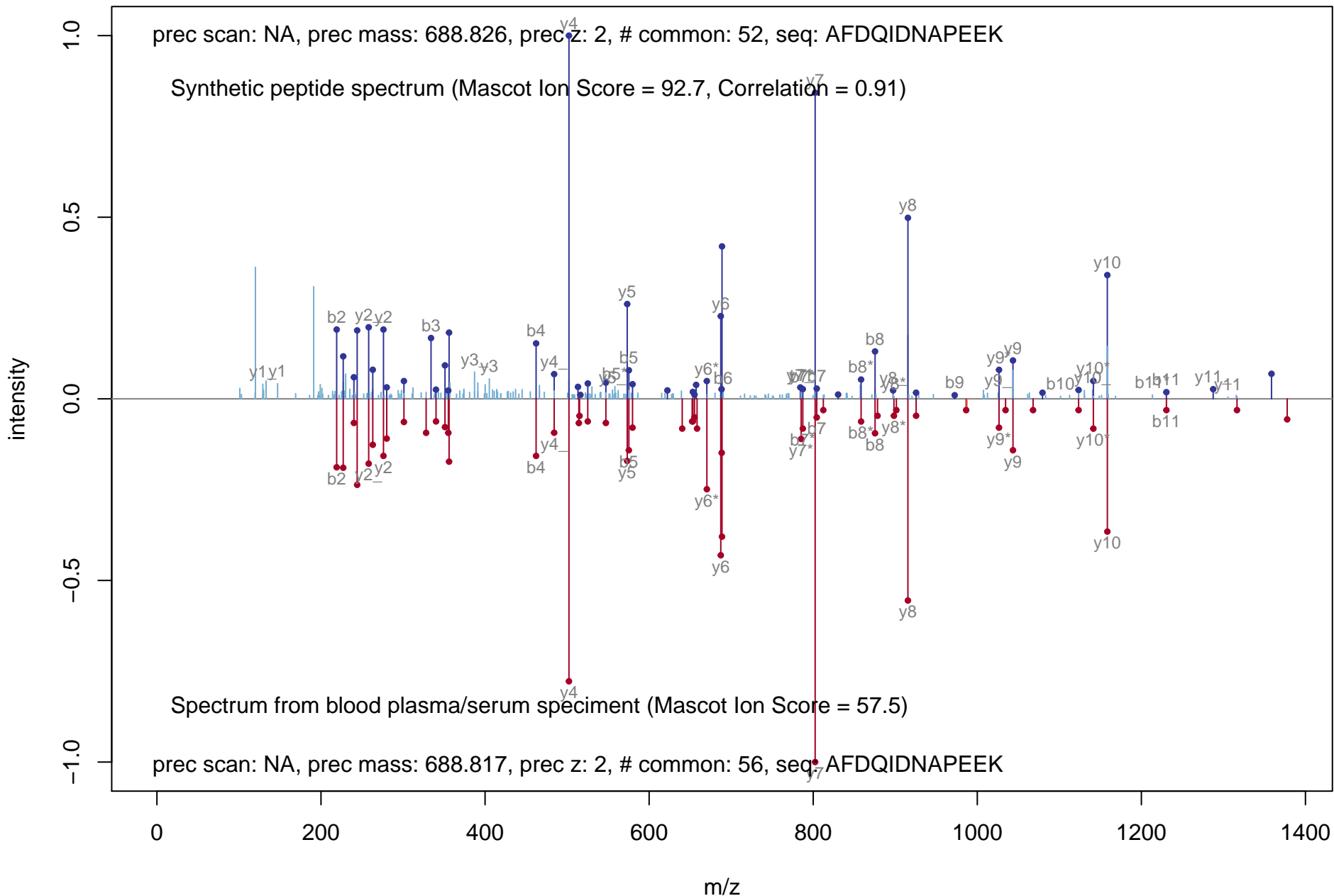

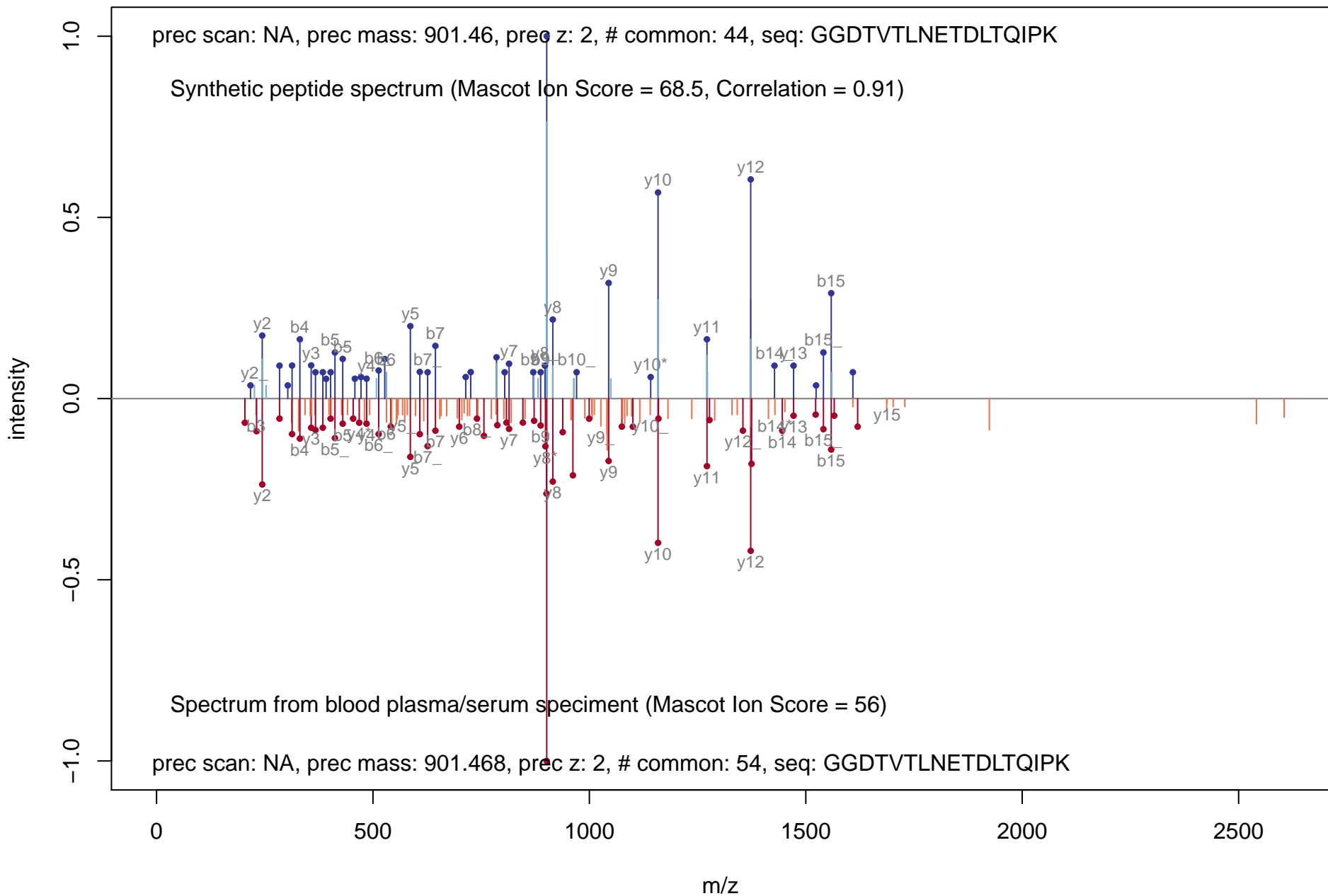

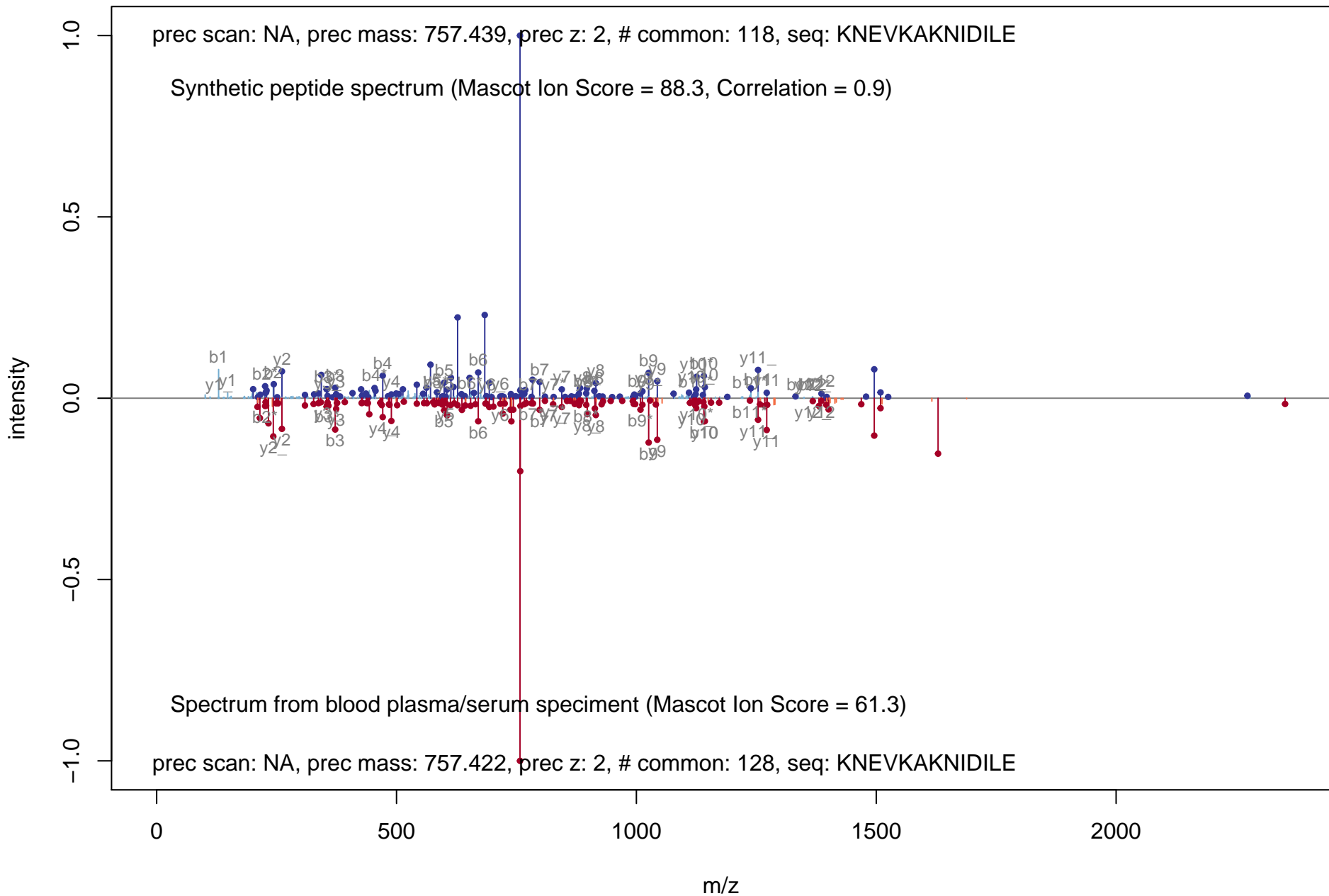

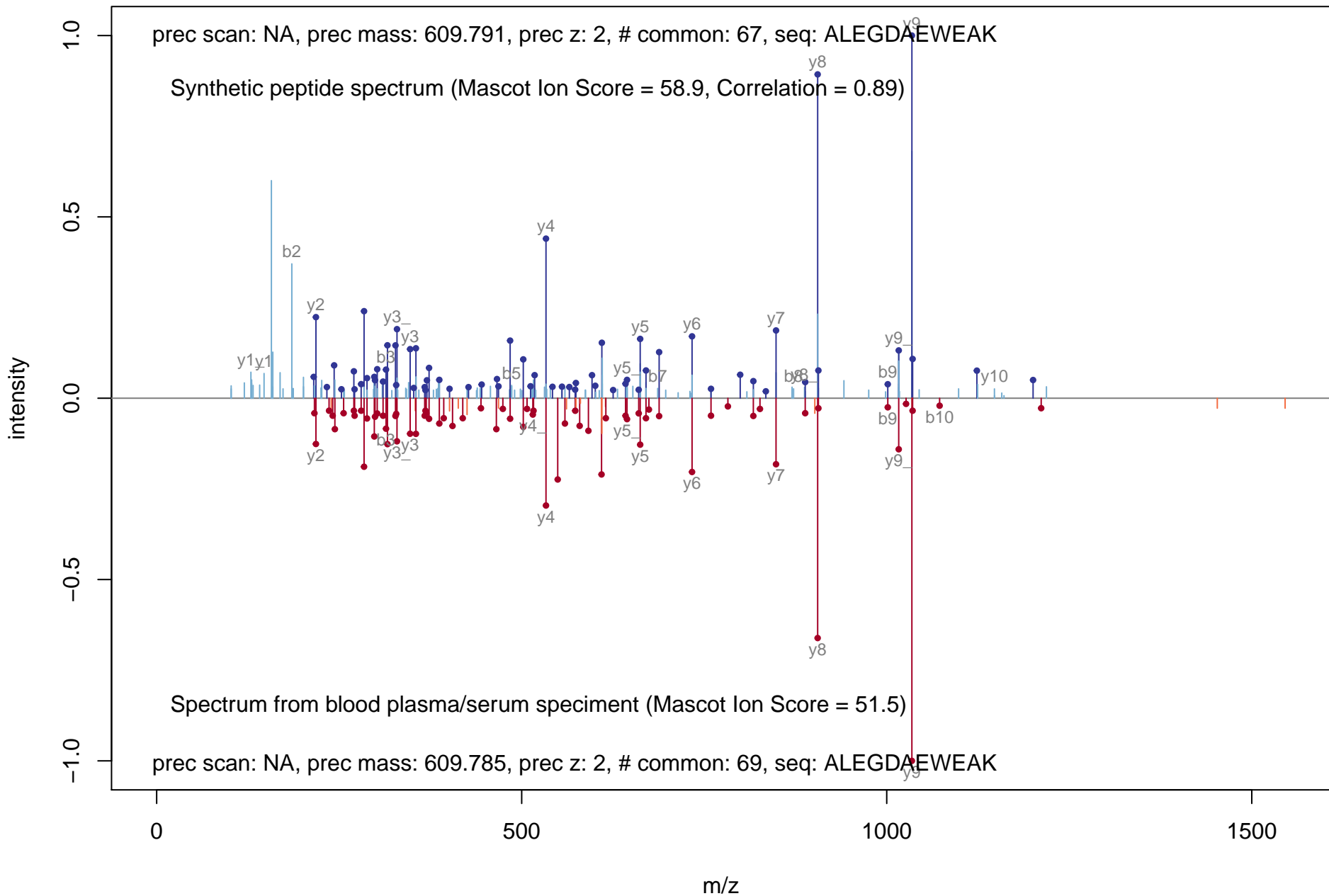

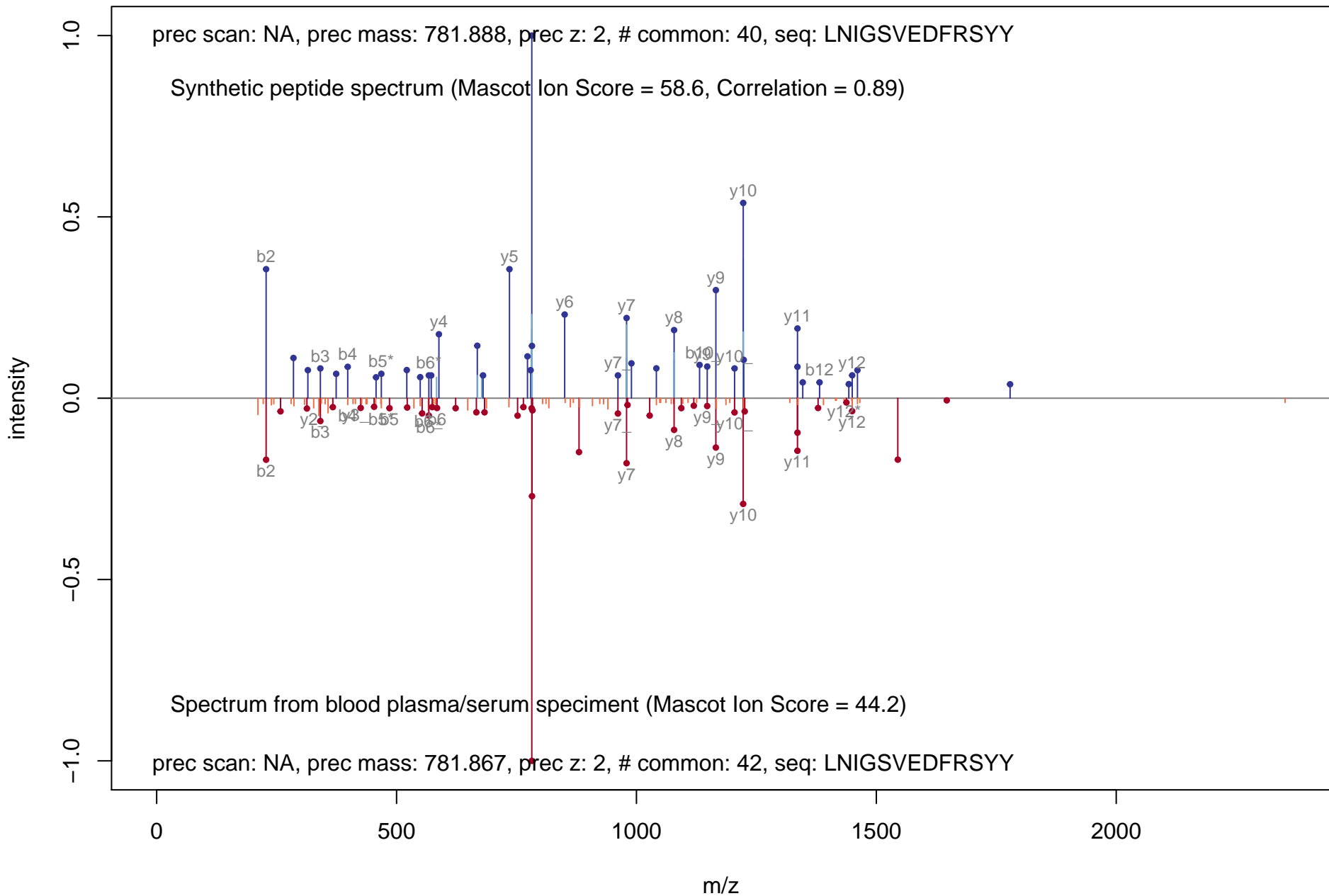

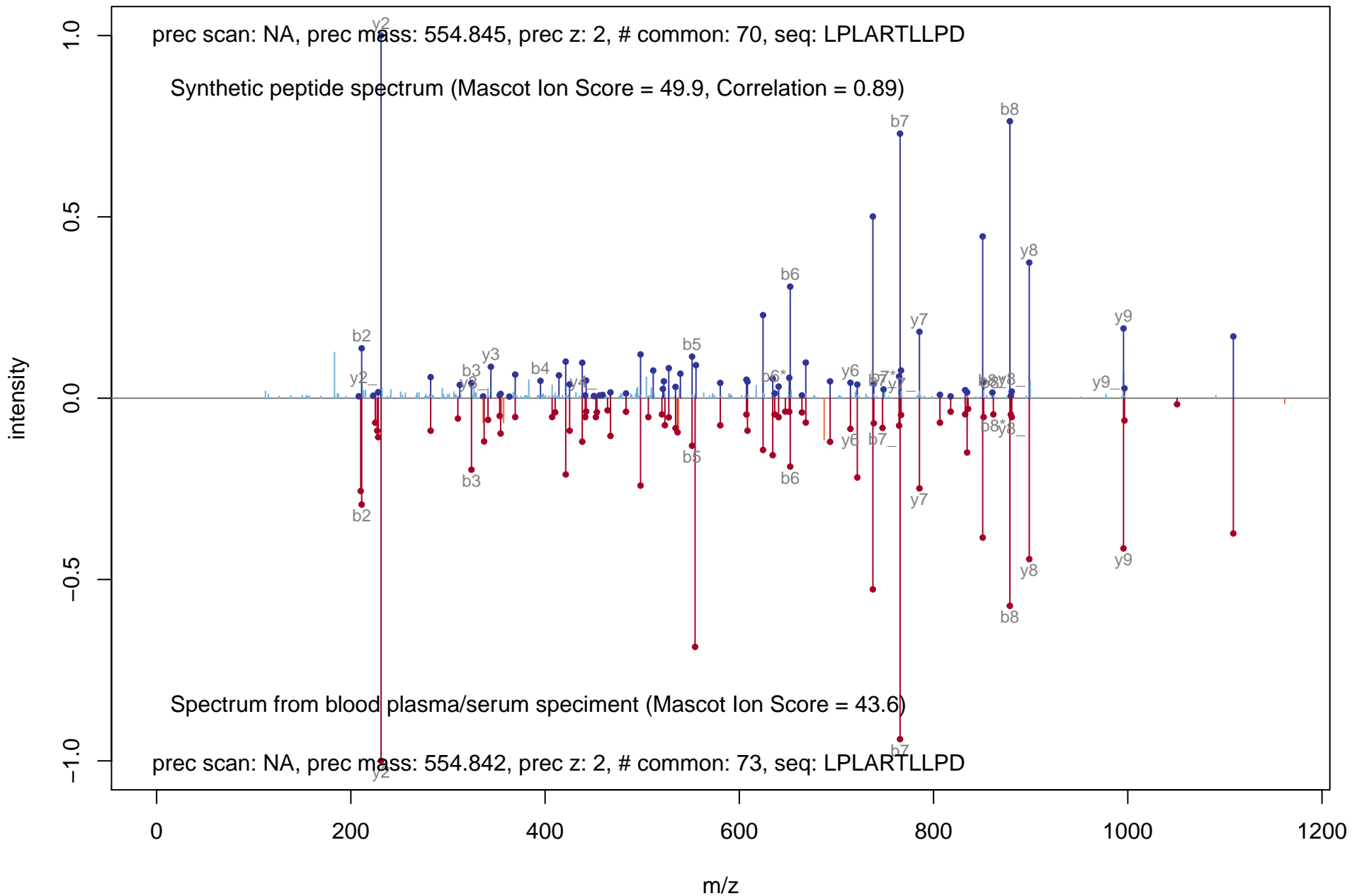

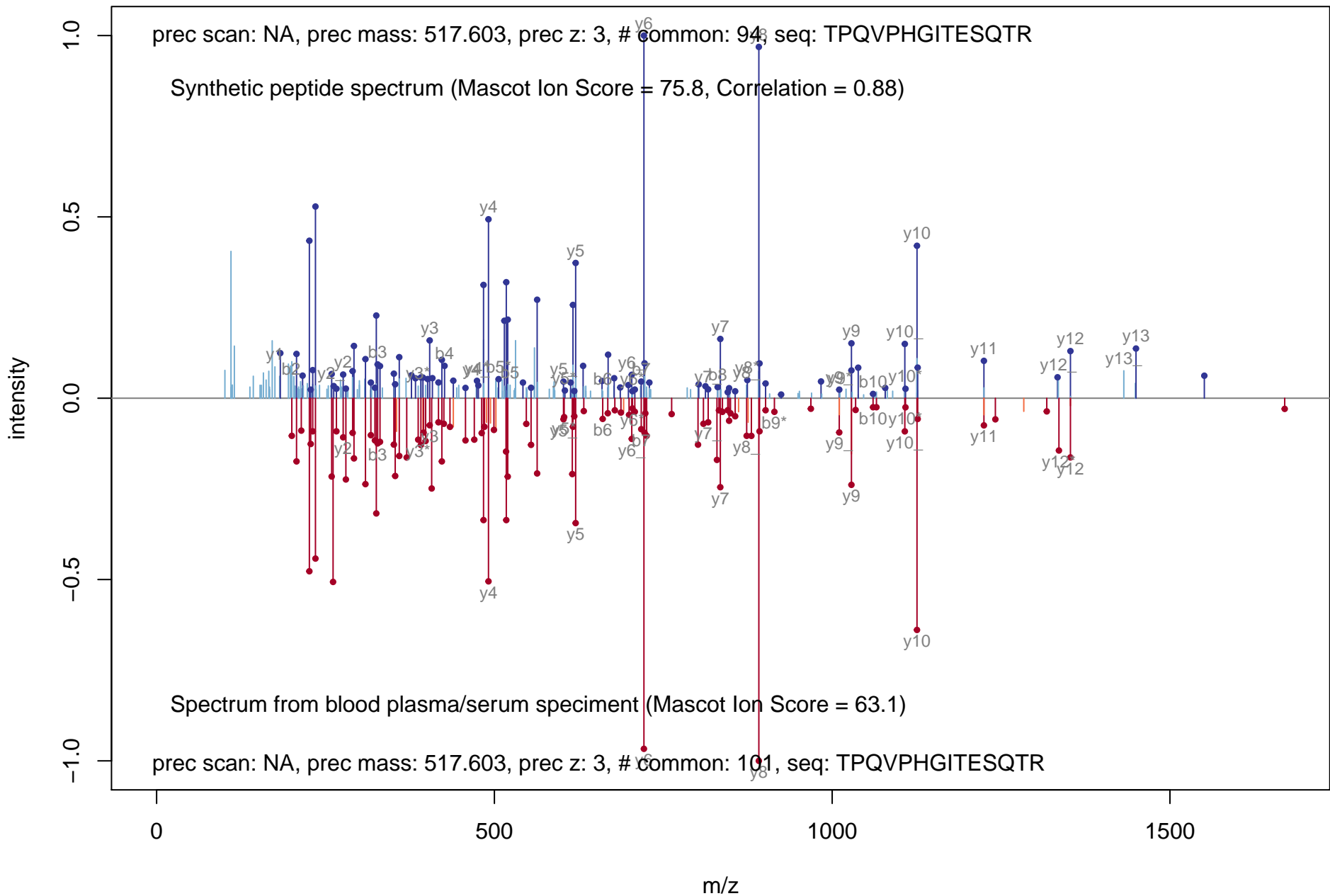

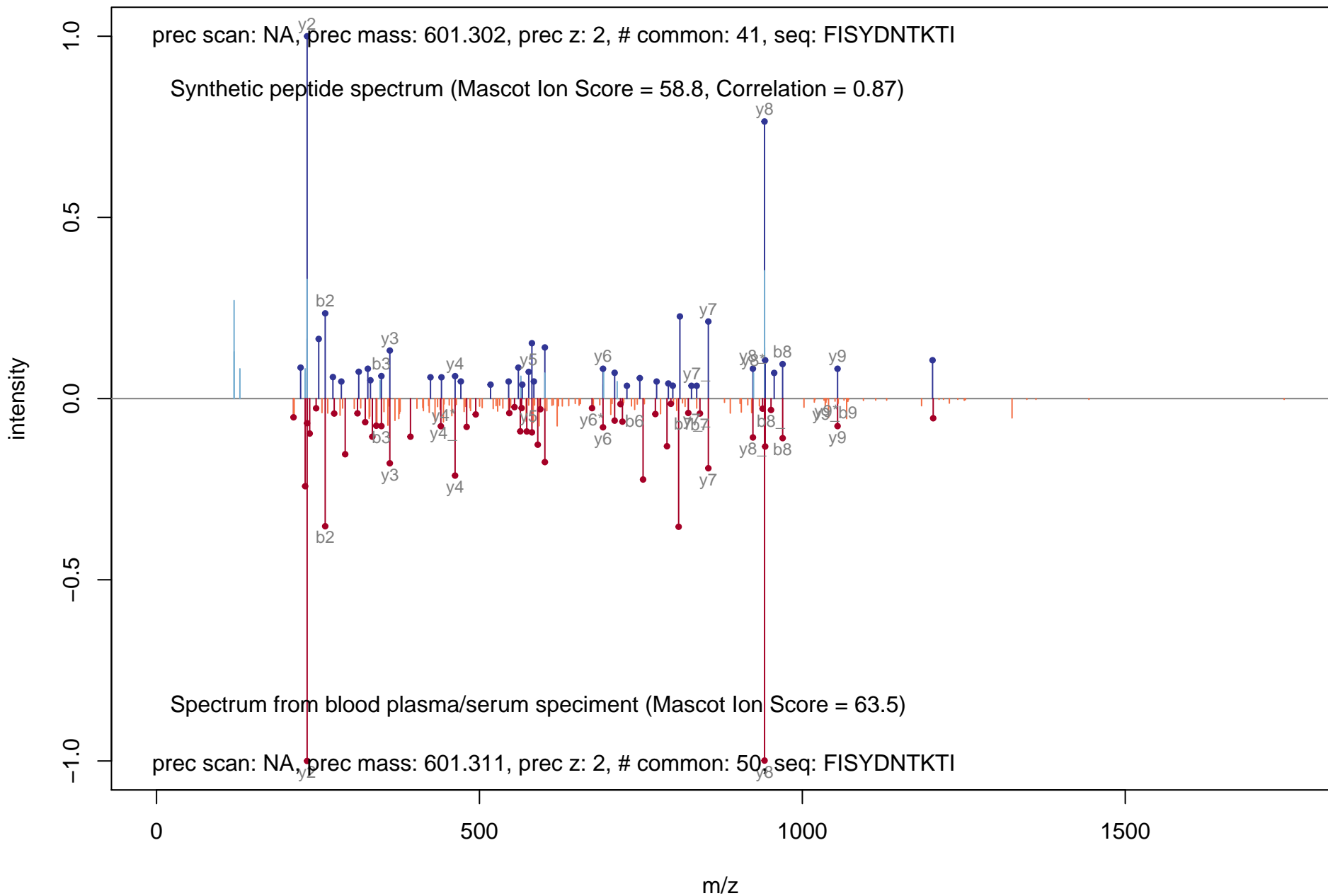

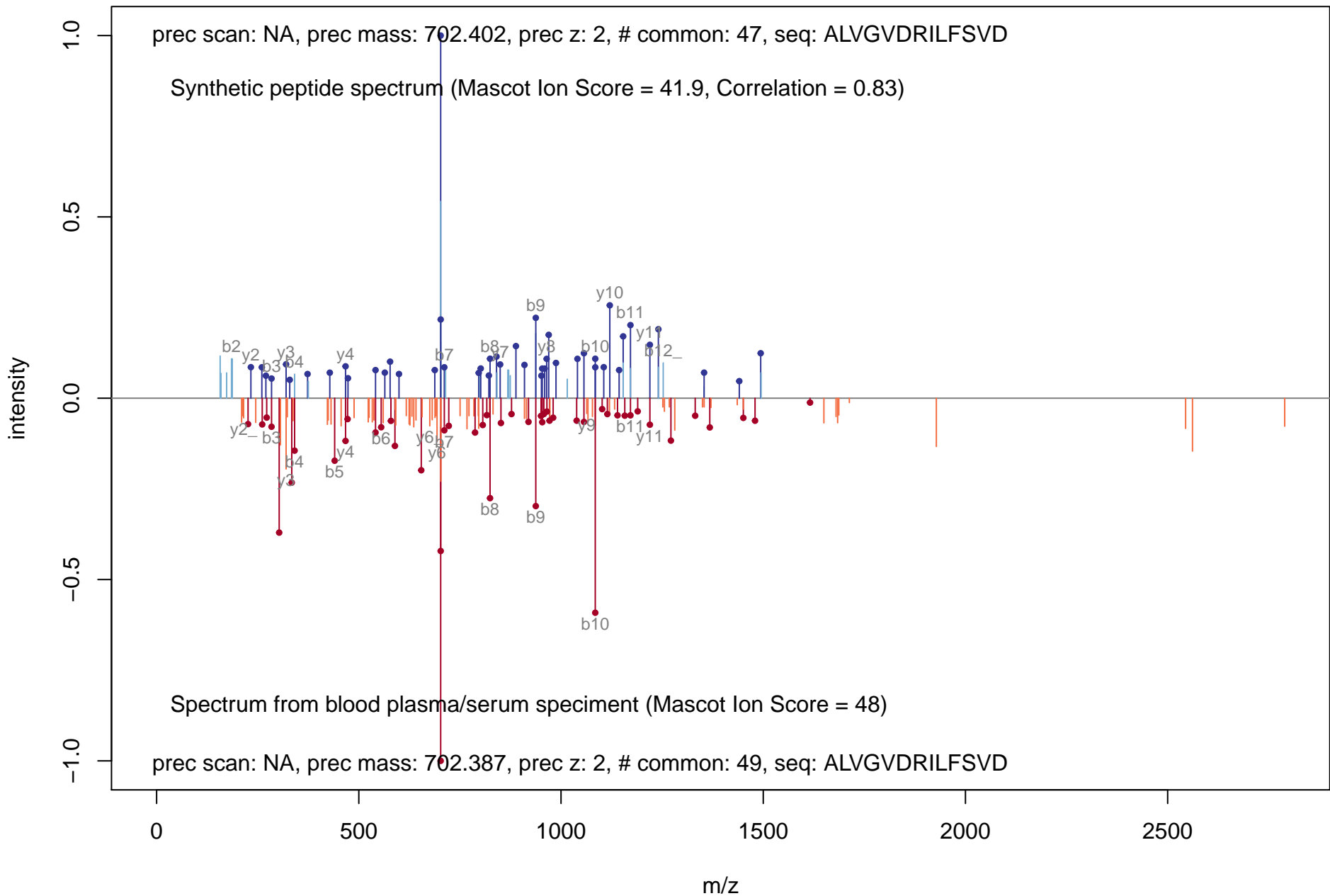

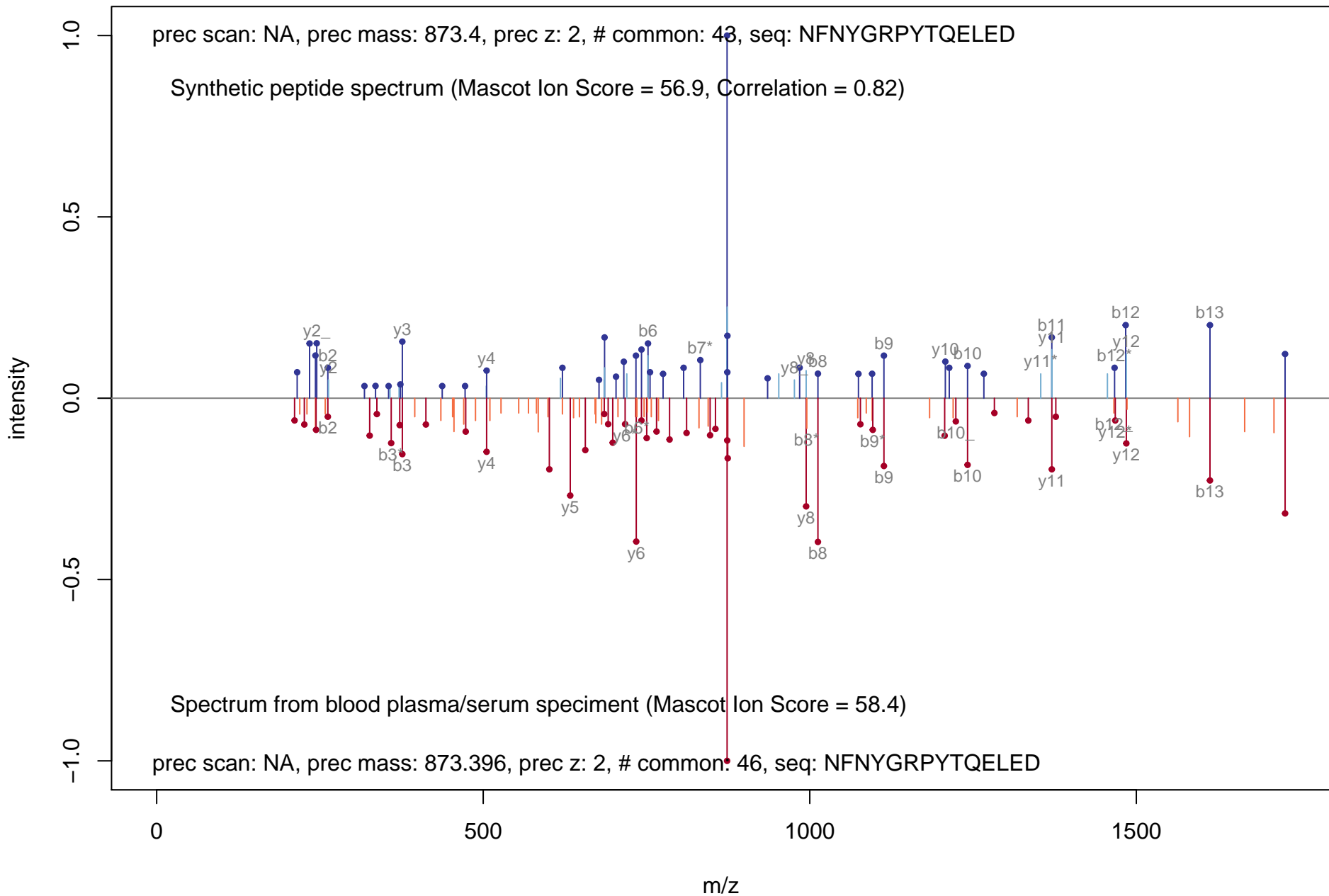

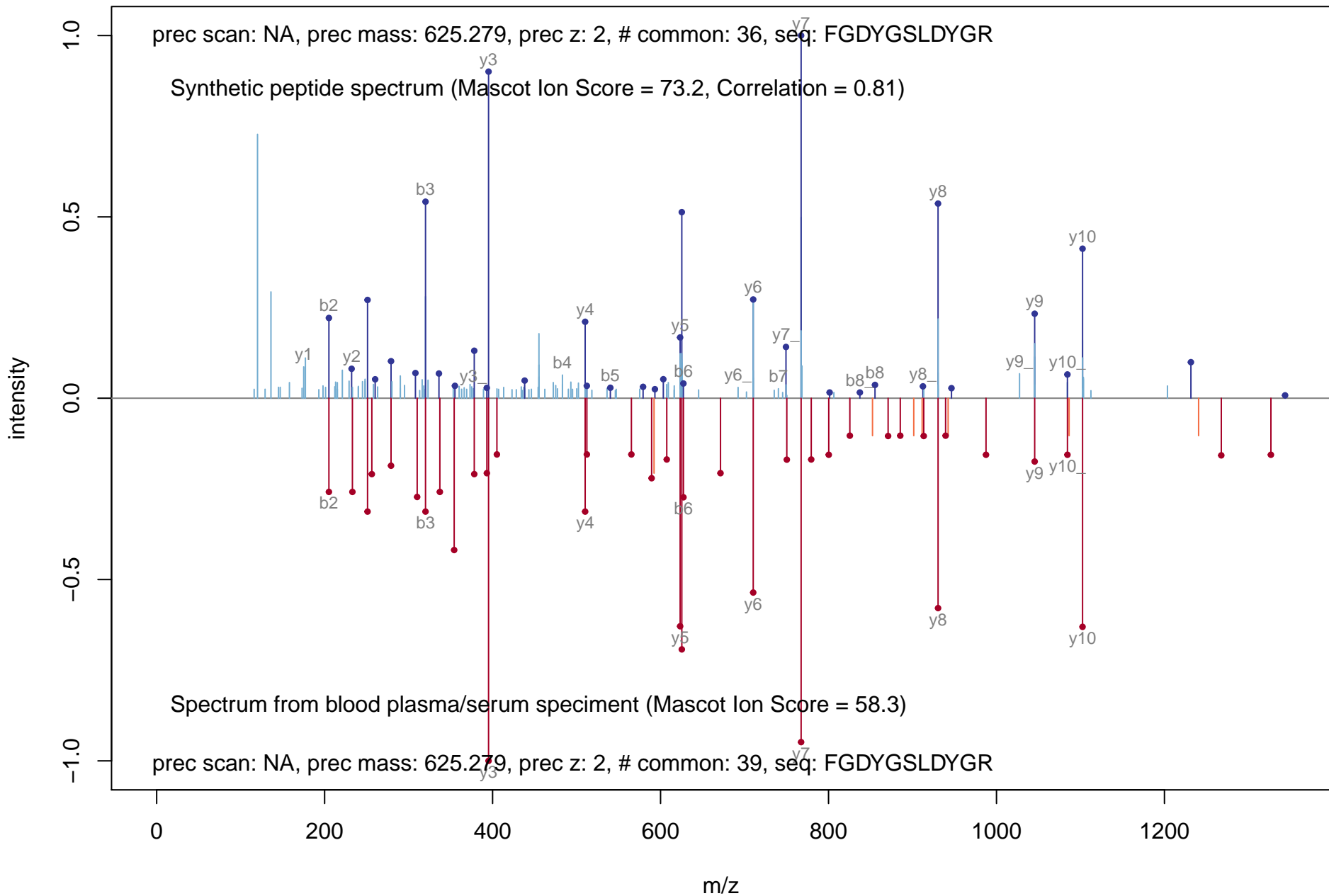

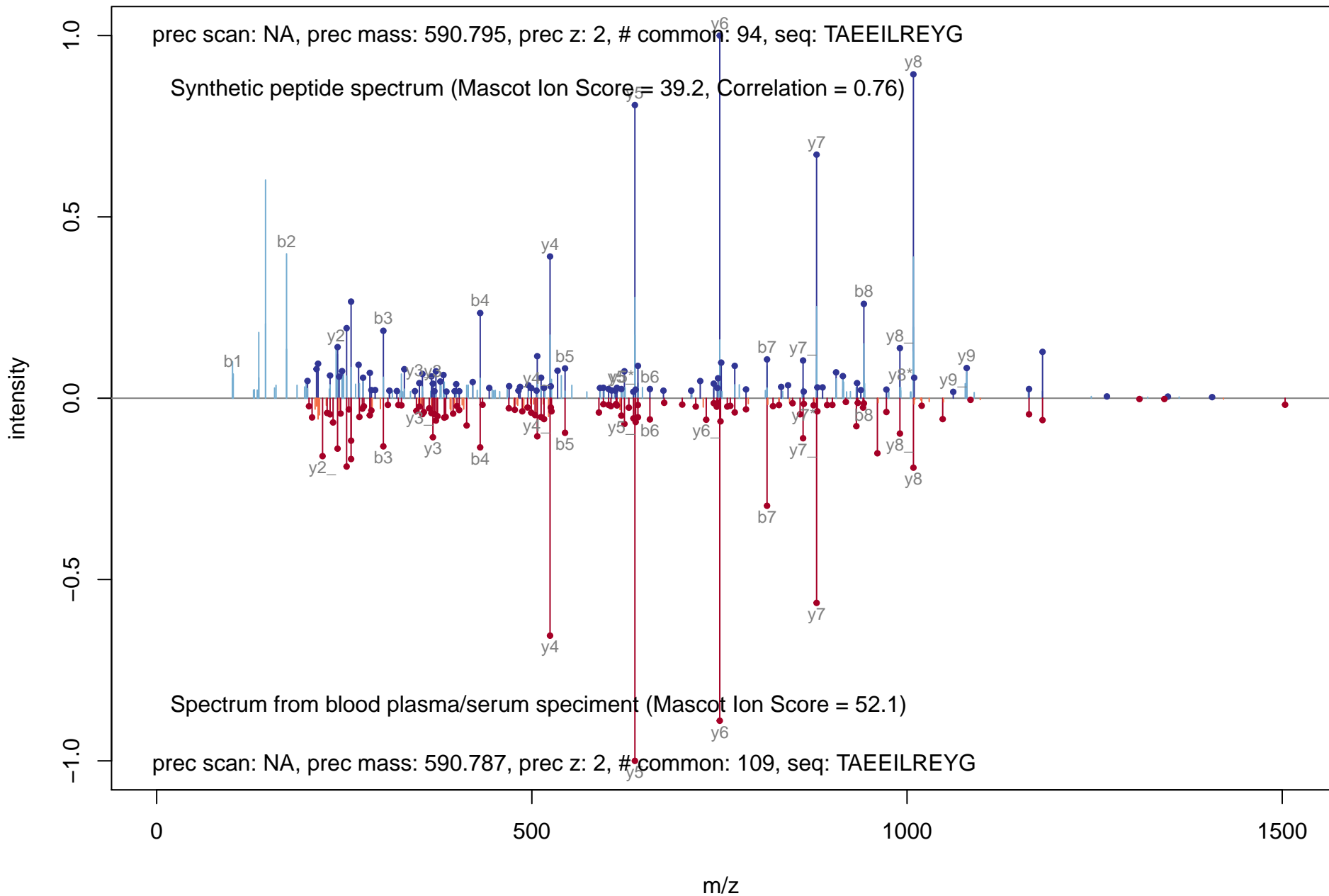

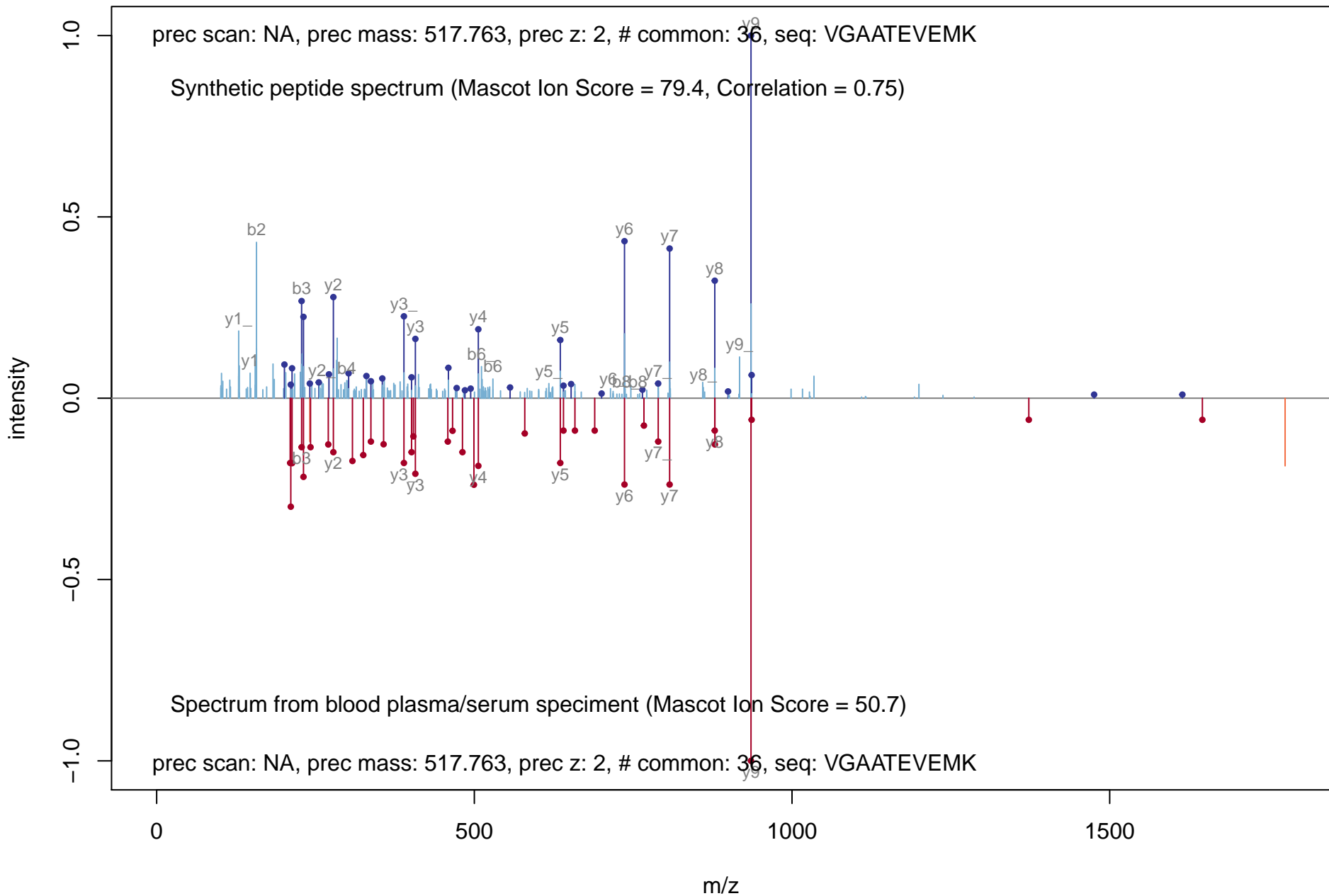

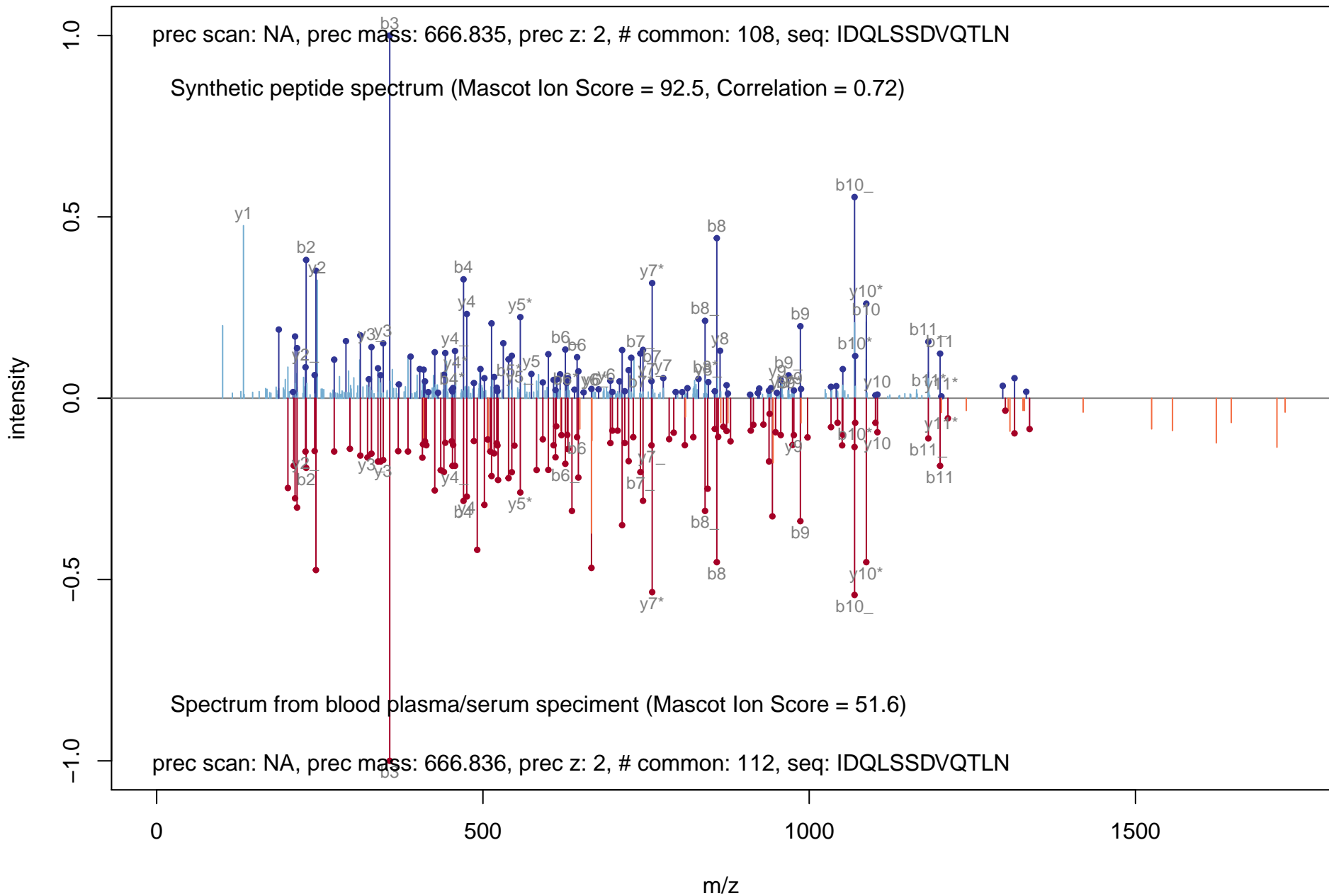

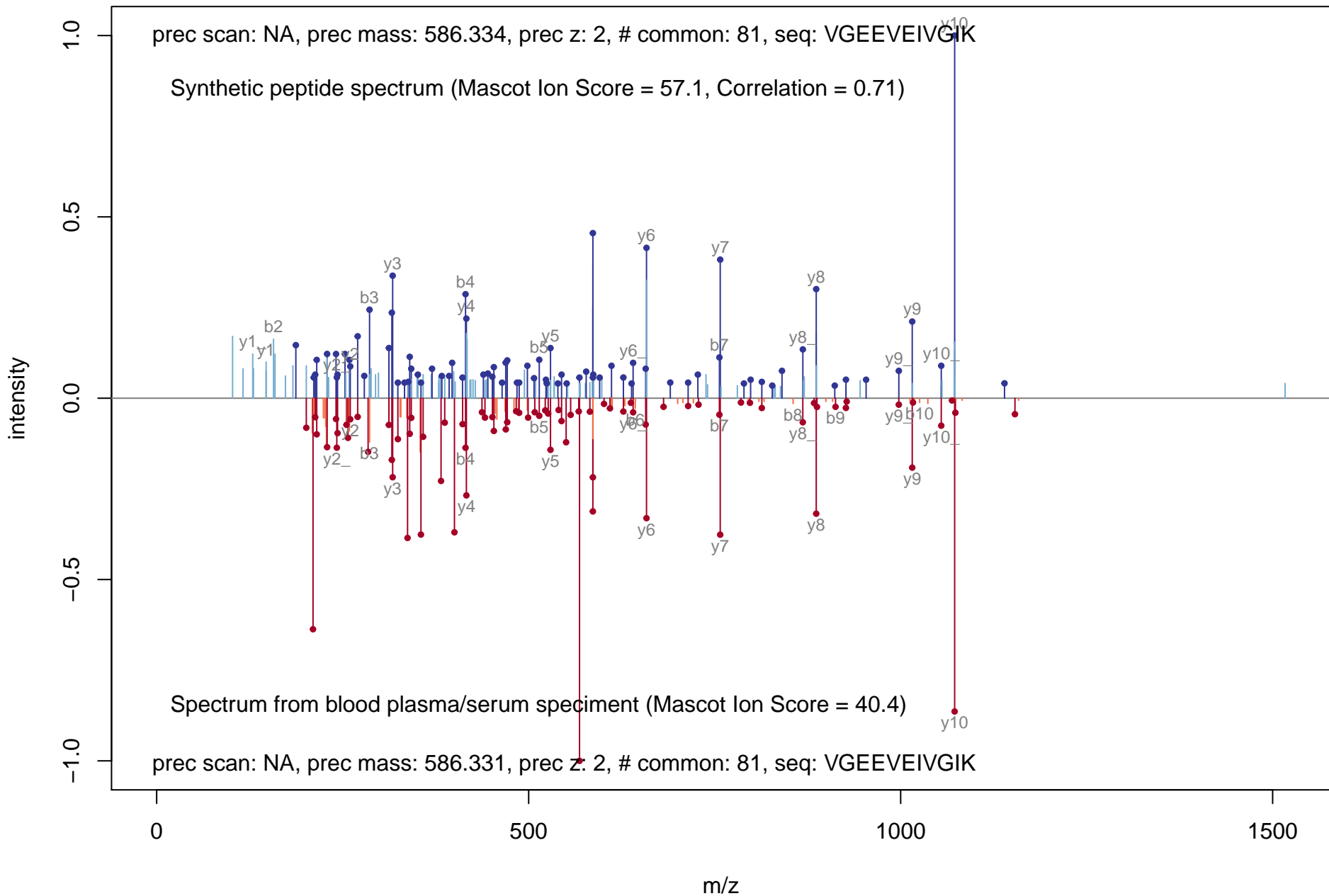

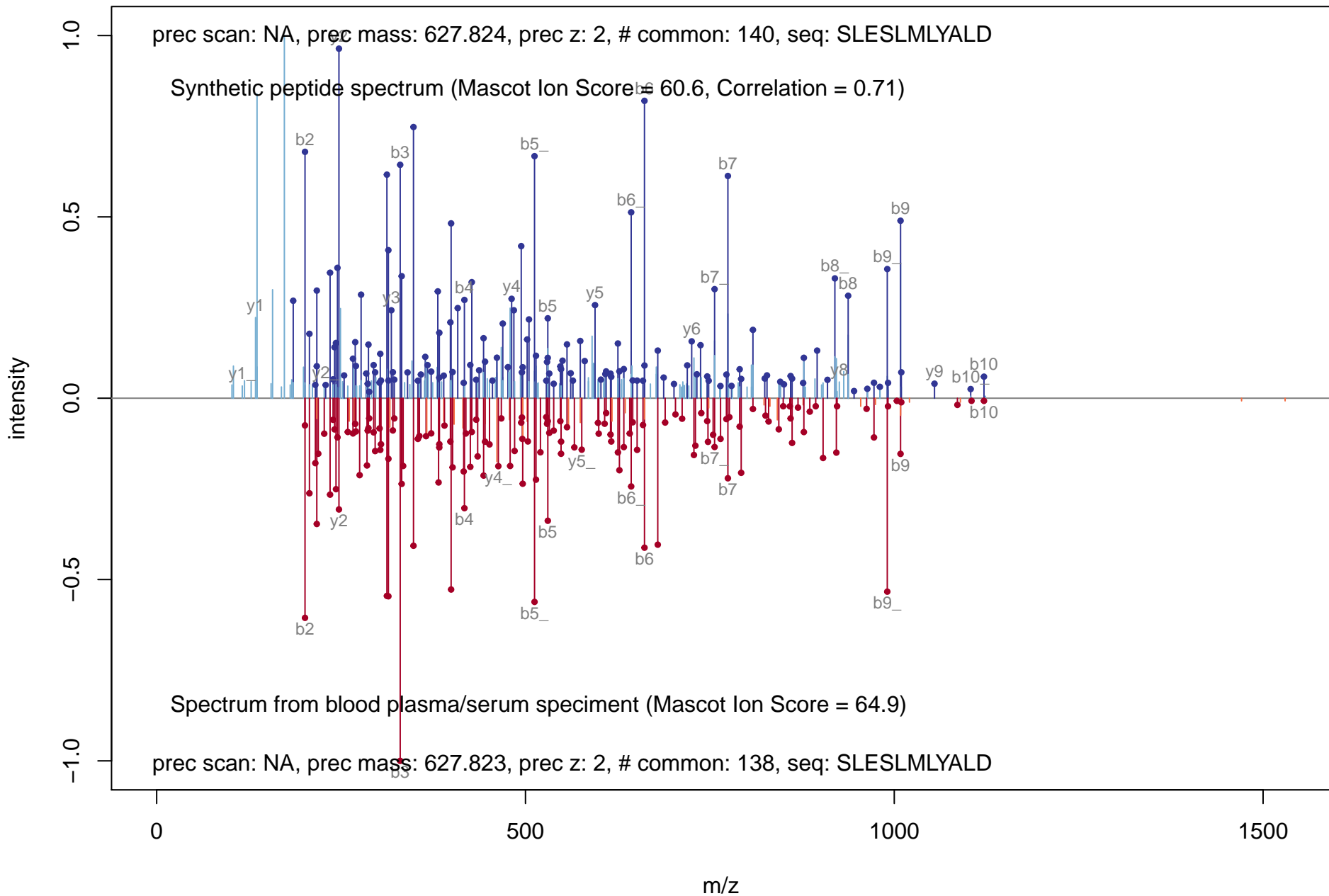

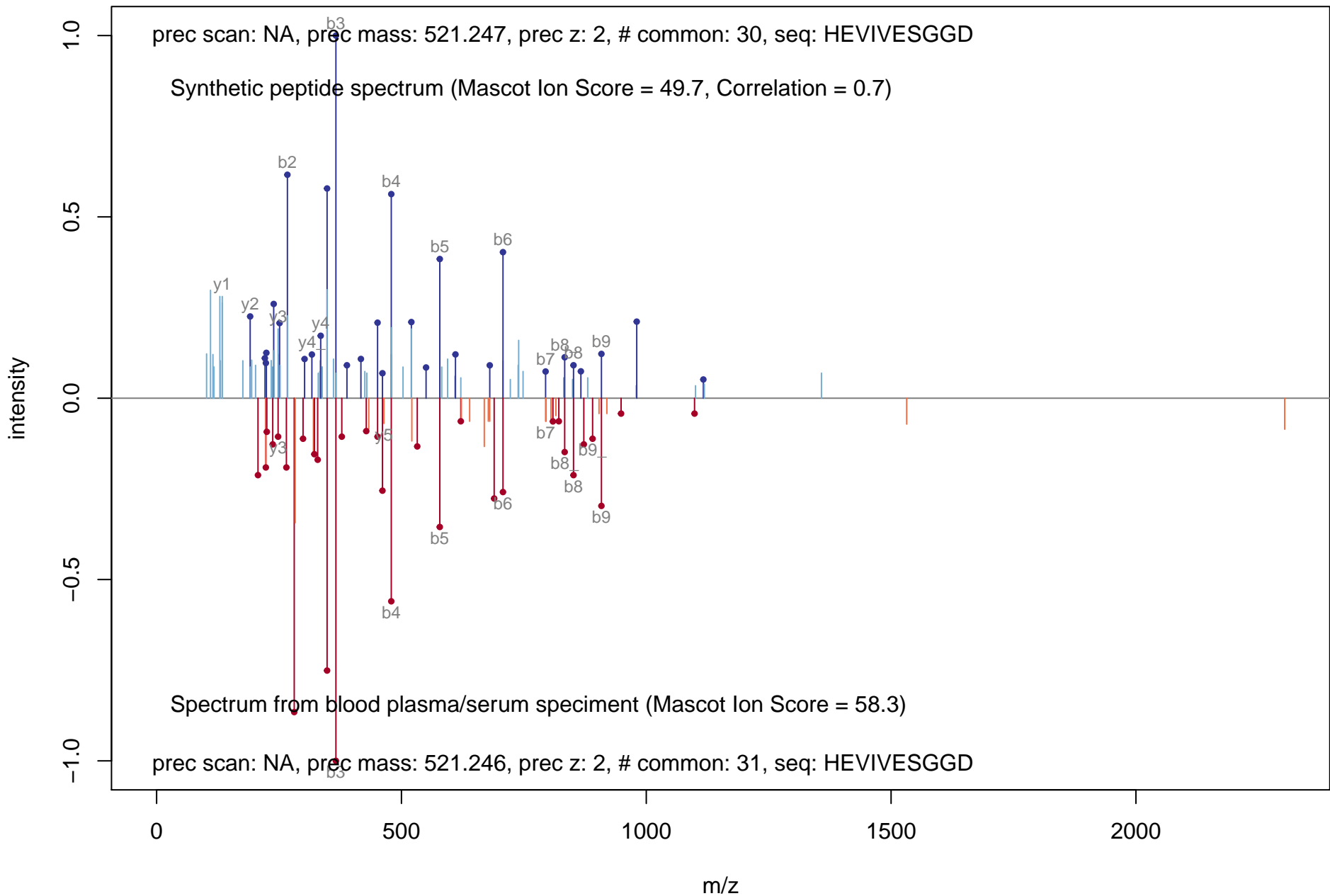

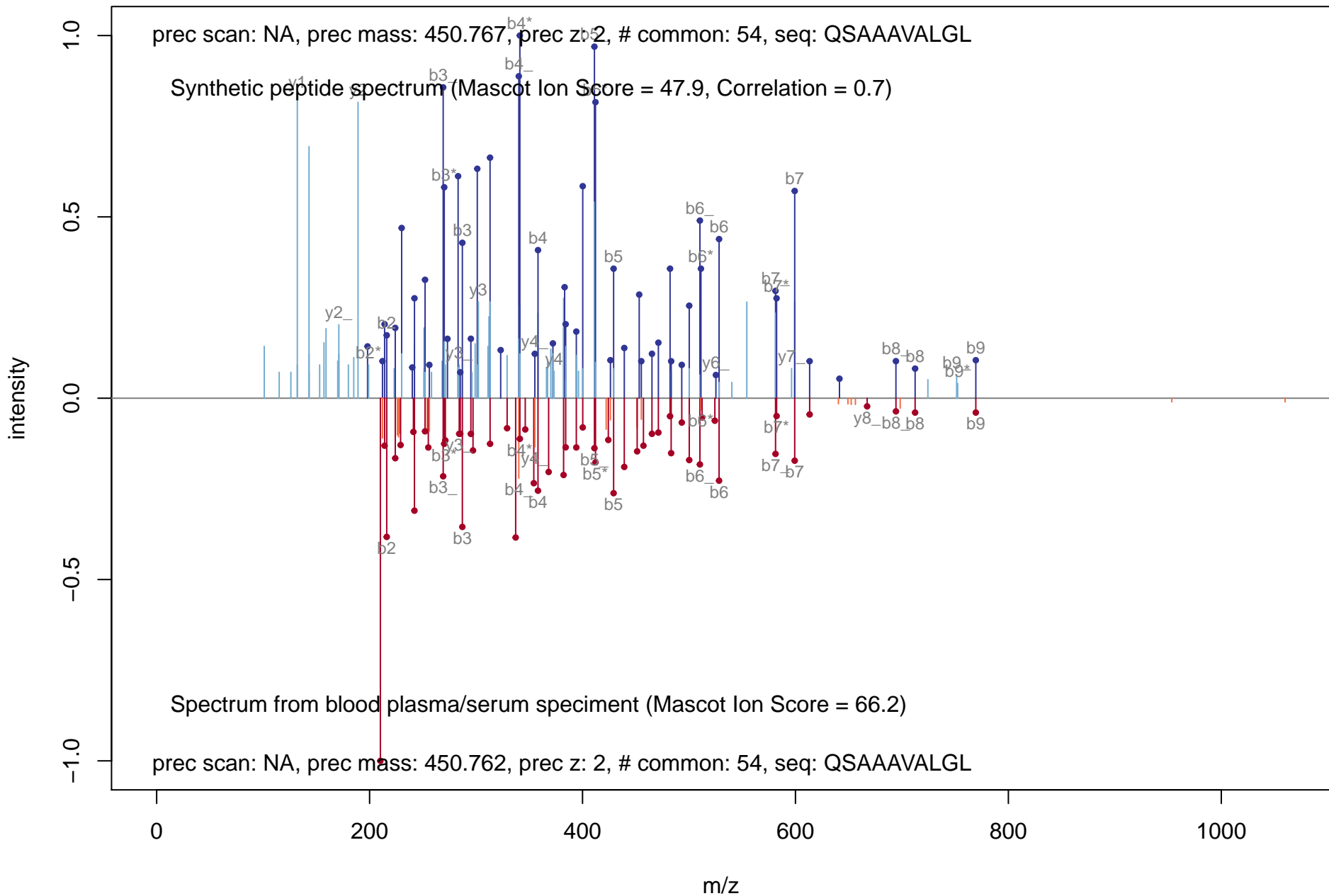

Supplement: Supplementary file 4 — Additional file 4. Related to Fig. 1. Comparison of 30 spectra identified in the blood plasma/serum samples and the spectra of synthetic peptides. b- and y-fragment peaks are labeled. Common peaks are shown in a slightly darker color. The estimates of Mascot Ion Score identification reliability are given. [file 12915_2024_1975_MOESM4_ESM.pdf]
